# Aqueous-Phase Hydrogenolysis of Glycerol over Re Promoted Ru Catalysts Encapuslated in Porous Silica Nanoparticles

**DOI:** 10.3390/nano8030153

**Published:** 2018-03-09

**Authors:** Kuo-Tseng Li, Ruey-Hsiang Yen

**Affiliations:** Department of Chemical Engineering, Tunghai University, 40704 Taichung, Taiwan; largestar790606@gmail.com

**Keywords:** Re-Ru@SiO_2_ nanoparticles, glycerol hydrogenolysis, 1,2-propanediol, 1,3-propanediol, diols, alcohols

## Abstract

Activity improvement of Ru-based catalysts is needed for efficient production of valuable chemicals from glycerol hydrogenolysis. In this work, a series of Re promoted Ru catalysts encapuslated in porous silica nanoparticles (denoted as Re-Ru@SiO_2_) were prepared by coating silica onto the surface of chemically reduced Ru-polyvinylpyrrolidone colloids, and were used to catalyze the conversion of glycerol to diols and alcohols in water. X-ray diffraction (XRD), scanning electron microscopy (SEM), transmission electron microscopy (TEM), nitrogen adsorption, X-ray photoelectron spectroscopy (XPS) and temperature-programmed reduction (TPR) were used to characterize these nanoparticles. Effects of Ru/Si atomic ratio, Re addition, glycerol and catalyst concentrations, reaction time, temperature, and hydrogen pressure were investigated. Re addition retarded the reduction of ruthenium oxide, but increased the catalyst reactivity for glycerol hydrogenolysis. Due to its greater Ru content, Re-Ru@ SiO_2_ showed much better activity (reacted at much lower temperature) and more yields of 1,2-propanediol and overall liquid-phase products than Re-Ru/SiO_2_ (prepared by conventional impregnation method) reported before. The rate of glycerol disappearance exhibited first-order dependence on glycerol concentration and hydrogen pressure, with an activation energy of 107.8 kJ/mol. The rate constant increased linearly with increasing Ru/Si atomic ratio and catalyst amount. The yield of overall liquid-phase products correlated well with glycerol conversion.

## 1. Introduction

**Figure 1:**
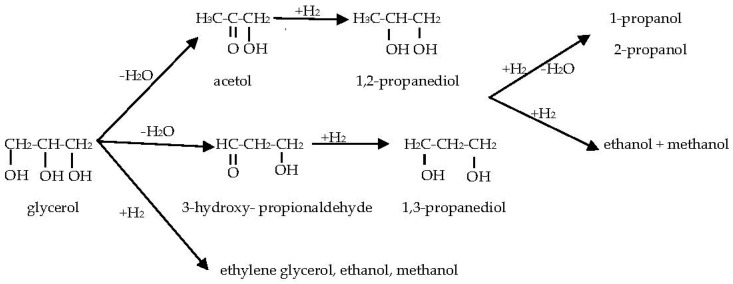
Reaction scheme of glycerol hydrogenolysis.

Glycerol is the co-product of biodiesel manufacturing and has become one of the top 12 building blocks of biorefinery [1,2]. The conversion of glycerol to other valuable chemicals is needed to solve its oversupply problem [3,4]. Several useful C1–C3 diols and alcohols can be produced from glycerol hydrogenolysis, via the reaction scheme shown in Figure 1 [5,6].

Among these diols/alcohols, 1,2-propanediol (1,2-PDO) is mainly used as a raw material for producing unsaturated polyester resins, antifreeze fluid, solvent, and preservatives. 1,3-propanediol (1,3-PDO) is used as the monomer for the production of polypropylene terephthalate (PPT), which is a biodegradable polyester and has great potential for use in carpet and textile manufacturing. 1-propanol (1-PrOH) is used mainly as a solvent, a printing ink, and a chemical intermediate for the production of n-propyl acetate. 2-propanol (2-PrOH) is used as a solvent. Ethylene glycol (EG) is mainly used as an antifreeze fluid and a raw material for polyethylene terephthalate (PET). Methanol is used for making formaldehyde, methyl tert-butyl ether (MTBE), biodiesel and di-methyl ether [7].

Ruthenium-based catalysts, including Ru/C with an ion-exchanged resin [8,9], Ru on various supports (SiO_2_, Al_2_O_3_, zeolites, graphite, carbon nanotubes, MCM-41) [5,10,11,12], Ru-Cu/TiO_2_ [13], Re-Ru on different supports (SiO_2_, Al_2_O_3_, carbon, ZrO_2_) [14,15] have been studied for catalyzing glycerol hydrogenolysis to produce diols/alcohols in the presence of high pressure hydrogen. Ru/SiO_2_ catalyst prepared with impregnation method showed low activity in the hydrogenolysis of glycerol [16]. Vasiliadou et al. obtained 20% glycerol conversion with 65% 1,2-PDO selectivity at 240 °C using a Ru/SiO_2_ catalyst prepared with wet impregnation method [5]. Ma and He obtained 51.7% glycerol conversion and 25% 1,2-PDO yield at 160 °C, 8 MPa and 8 h, using a Re-Ru/SiO_2_ catalyst prepared by impregnating SiO_2_ powder with aqueous solution of RuCl_3_·4H_2_O and HReO_4_ [15]. Therefore, activity improvement of Ru-based catalysts is needed for efficient production of diols/alcohols from glycerol hydrogenolysis.

We have prepared palladium core-porous silica shell-nanoparticles (denoted as Pd@SiO_2_) for catalyzing 4-carboxybenzaldehyde hydrogenation [17] and prepared copper core-porous silica shell-nanoparticles (denoted as Cu@SiO_2_) for catalyzing glycerol hydrogenolysis in methanol [18]. These nanoparticles exhibited better performances than the corresponding catalysts prepared by conventional impregnation methods. In addition, palladium nanoparticels encapsulated in porous silica shell have been proved to be highly stable for CO oxidation due to the sintering prevention effect of silica shell [19]. Although Cu@SiO_2_ nanoparticles had 96.5% 1,2-PDO yield in methanol at 200 °C, they exhibited very low activity for catalyzing glycerol hydrogenolysis in water. It is desirable to use environmental friendly solvent–water- and to decrease the reaction temperature for glycerol hydrogenolysis. In addition, the use of Ru-based catalysts for glycerol hydrogenolysis can produce several useful C1–C3 diols and alcohols simultaneously, as mentioned above.

In this work, glycerol hydrogenolysis was carried out in aqueous phase using Ru catalysts encapuslated in porous silica nanoparticles (denoted as Ru@SiO_2_) promoted with rhenium oxide. The Re-Ru@SiO_2_ catalysts exhibited very high activity at 130 °C, which was significantly lower than that (160 °C) used by Ma and He [15]. The use of lower reaction temperature resulted in greater yields of 1,2-propanediol and overall liquid-phase products.

## 2. Results and Discussion

### 2.1. Catalyst Characterization

**Figure 2:**
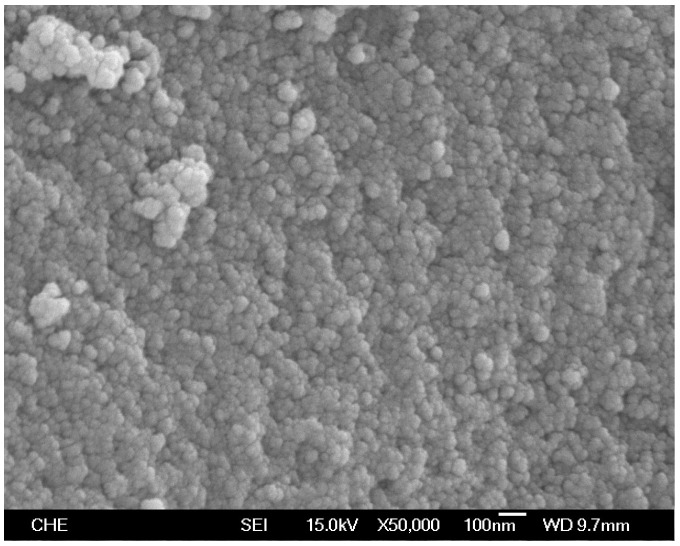
Scanning electron micrograph of Re-Ru@SiO_2_ particles with a Ru/Si atomic ratio of 0.4.

**Figure 3:**
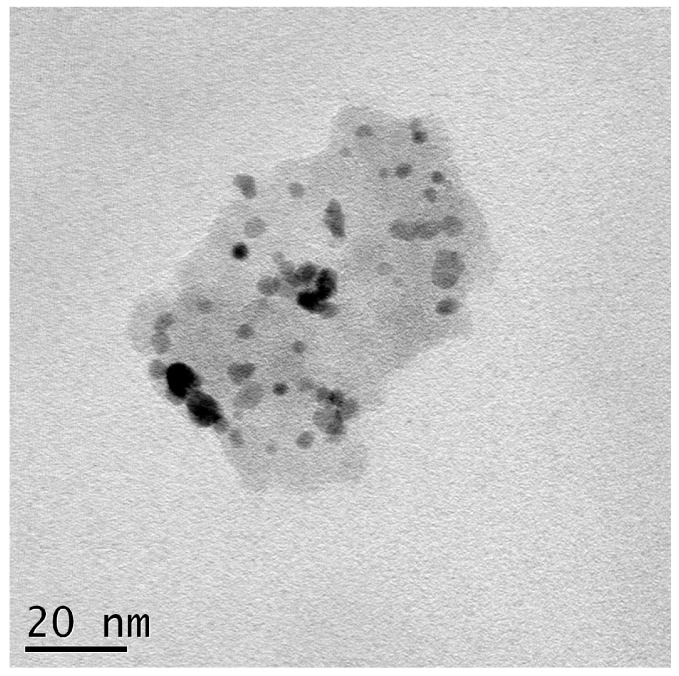
Transmission electron micrograph of Ru@SiO_2_ particles with a Ru/Si atomic ratio of 0.4.

Ru@SiO_2_ and Re-Ru@SiO_2_ nanoparticles with five different Ru/Si atomic ratios (in a range of 0.1–1.6) were prepared. The Re-Ru@SiO_2_ nanoparticles with Ru/Si atomic ratios of 0.1, 0.2, 0.4, 0.8 and 1.6 were denoted as Re-01RuSi, Re-02RuSi, Re-04RuSi, Re-08RuSi, and Re-16RuSi, respectively. Figure 2 shows a scanning electron micrograph of a Re-04RuSi sample, indicating that the particles have a size of 40–60 nm. Figure 3 shows a transmission electron micrograph of calcined Ru@SiO_2_ sample with a Ru/Si atomic ratio of 0.4, indicating that small ruthenium nanoparticles with a variety of sizes are encapsulated in silica.

**Figure 4:**
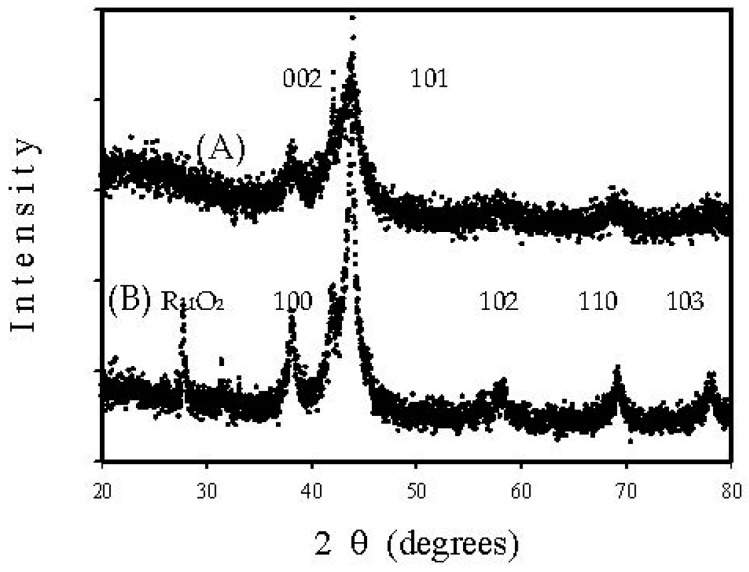
X-ray diffraction (XRD) patterns of reduced Re-Ru@SiO_2_ particles with Ru/Si atomic ratios of (A) 0.4 and (B) 0.8.

Figure 4 displays XRD patterns of (A) Re-04RuSi and (B) Re-08RuSi samples, which were reduced with 5% hydrogen in argon at a heating rate of 1 °C/min to 200 °C, and then maintained at 200 °C for 4 h. The patterns exhibit the characteristic peaks of finely divided metallic ruthenium at 2θ = 38°, 42°, 44°, 58°, 69° and 78°, which correspond to the reflection peaks of Ru(100), Ru(002), Ru(101), Ru(102), Ru(110) and Ru(103), respectively [20]. Peak intensity of profile (A) is much smaller than that of profile (B), suggesting that crystal size of Re-04RuSi is much smaller than that of Re-08RuSi. The existence of RuO_2_ diffraction peak (2θ = 28°) in profile (B) suggests that Re-08RuSi sample is not completely reduced during the reduction step at 200 °C. Peaks of Re species are not observed in Figure 4, suggesting that Re species is highly dispersed [16].

**Figure 5:**
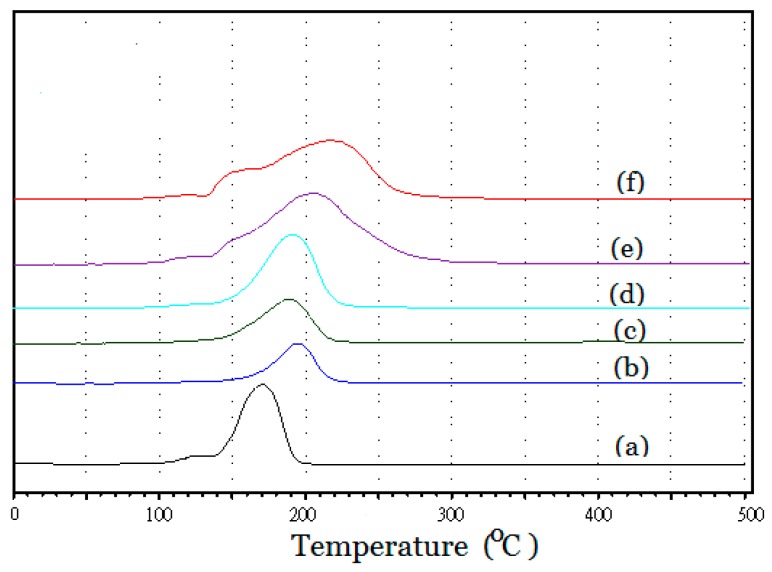
Temperature-programmed reduction (TPR) profiles of (a) Ru@SiO_2_ core-shell-particles with a Ru/Si atomic ratio of 0.4 (without Re addition) and Re-Ru@SiO_2_ core-shell-particles with Ru/Si atomic ratios of (b) 0.1, (c) 0.2, (d) 0.4, (e) 0.8 and (f) 1.6.

Catalyst reducibility was measured with a temperature-programmed reduction method using hydrogen as the reductant. Figure 5 compares the TPR profiles of (a) 04RuSi (without Re addition), (b) Re-01RuSi, (c) Re-02RuSi, (d) Re-04RuSi, (e) Re-08RuSi and (f) Re-16RuSi, indicating that RuO_2_ in these samples can be reduced completely below 350 °C and the reduction peak area increases with increasing Ru/Si atomic ratio.

Profile (a) has a reduction peak at 170 °C, which is essentially the same as the reduction peak temperature (167 °C) reported for a Ru/SiO_2_ catalyst prepared with the incipient wetness technique using Ru(NO)(NO)_3_ solution [21], corresponding to the reduction of RuO_2_ formed after catalyst calcination. Comparisons of profile (a) and profile (d) indicate that Re addition retards the reduction of ruthenium oxide because peak reduction temperature (T_max_) increases from 170 °C in profile (a) to 193 °C in profile (d). This implies an interaction effect between the Ru and Re particles which shifted the reduction peak to the higher temperature [22]. Shozi et al. [22] also found that the presence of rhenium increased the reduction temperature of Cu-ZnO catalyst.

Profiles (e) and (f) in Figure 5 have large and broad reduction peaks, which is due to their greater Ru content. T_max_ of profiles (e) and (f) are 205 °C and 222 °C, respectively, which are higher than those in profiles (b) to (d) (in a range of 190 °C to 200 °C). It was reported in the literature that the reduction temperature of unsupported RuO_2_ is 217 °C [23]. The higher reduction temperatures of profiles (e) and (f) suggest that some RuO_2_ enclosed in Re-08RuSi and Re-16RuSi nanoparticles do not contact with silica shell, and they have a reduction temperature similar to that of unsupported Ru species.

Pore size distribution analyses of Re-04RuSi and Re-08RuSi samples indicated that these particles were mesoporous materials with a major pore diameter of 3.8 nm. Pore volume decreased with increasing Ru/Si atomic ratio (pore volumes were 0.6, 0.4, 0.35 and 0.33 cm^3^/g for Ru/Si atomic ratios of 0.2, 0.4, 0.8, and 1.6, respectively), suggesting that most pores were generated in the silica shell due to burning out of organic material (PVP). It is known that fine pores in the range of 1 to 10 nm in radius account for most of the surface area [24], therefore, the decrease of pore volume with increasing Ru/Si ratio resulted in the decrease of catalyst surface area (surface areas were 405.2, 353.6, 230.9 and 188.3 m^2^/g for Ru/Si atomic ratios of 0.2, 0.4, 0.8, and 1.6, respectively). Scanning electron micrograph images of catalysts indicated that catalyst particle size decreased continuously with increasing Ru/Si atomic ratio. This is contrary to the results of surface area measurements (surface area decreased with increasing Ru/Si atomic ratio), indicating that internal surface is more important than the external surface for determining the total surface area. The addition of Re species on Ru@SiO_2_ resulted in the significant decrease of surface area. For example, surface areas decreased from 420 m^2^/g (without Re addition) to 353.6 m^2^/g (with Re addition) when Ru/Si atomic ratio = 0.4.

**Figure 6:**
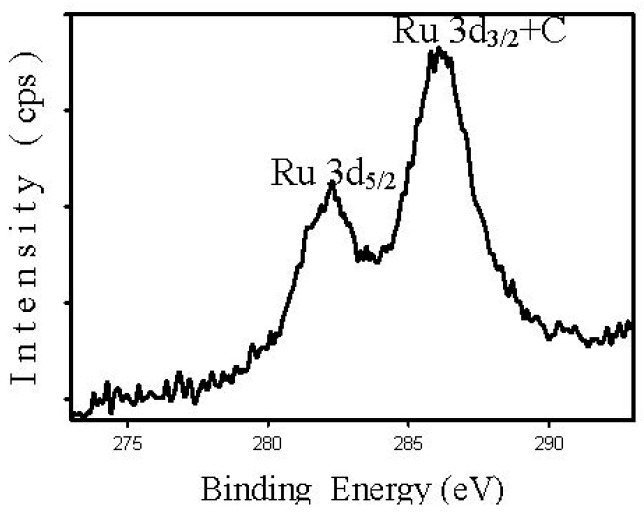
Ru 3d XPS spectra of the Re-Ru@SiO_2_ nanoparticles.

Figure 6 shows the Ru 3d spectrum for Re-Ru@SiO_2_ nanoparticles, which has one set of doublets with a spin-orbit splitting of 3.8 eV. The first peak of Ru 3d is located at about 282.3 eV, which is similar to that (282.4 eV) reported before for a Ru/SiO_2_ catalyst [25], suggesting that Re addition does not modify the electronic properties of ruthenium.

**Figure 7:**
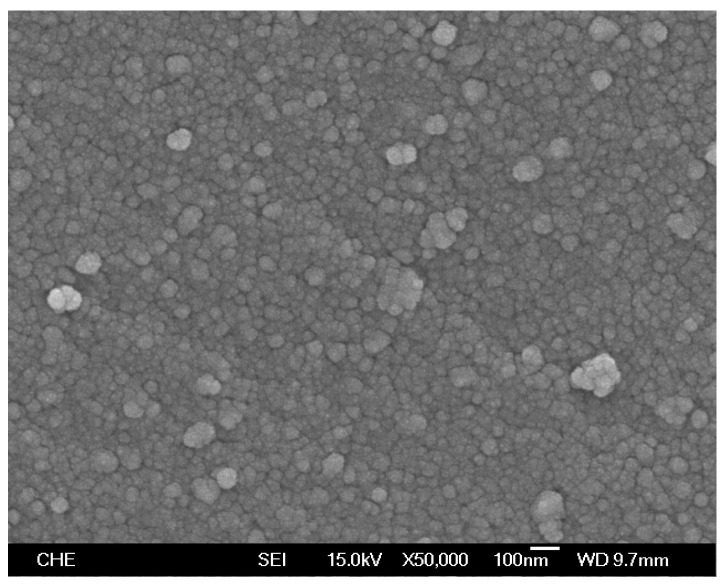
Scanning electron micrograph of spent Re-04RuSi catalyst.

**Figure 8:**
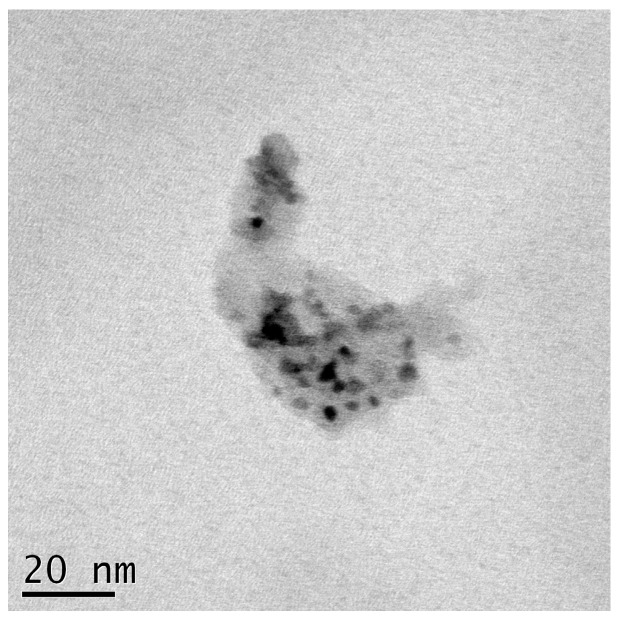
Transmission electron micrograph of spent Re-04RuSi catalyst.

Figure 7 and Figure 8 show a scanning electron micrograph and a transmission electron micrograph of used Re-04Ru@SiO_2_ nanoparticles, respectively. A comparison of Figure 7 and Figure 2 indicates that particle size does not change significantly after hydrogenolysis. Figure 8 shows that small ruthenium nanoparticles with a variety of sizes remain highly dispersed in silica, indicating that the catalyst is stable under the glycerol hydrogenolysis conditions.

### 2.2. Hydrogenolysis of Glycerol

Glycerol hydrogenolysis was carried out with five Re-Ru@SiO_2_ catalysts mentioned above, five reaction temperatures (110–160 °C), six reaction times (2–12 h), five hydrogen pressure (0–1100 psig), three catalyst amounts (0.315–1.26 g) and five glycerol concentrations (10–50 wt %). The effects of these variables on glycerol conversion and diol/alcohol yields or selectivities were investigated.

**Figure 9:**
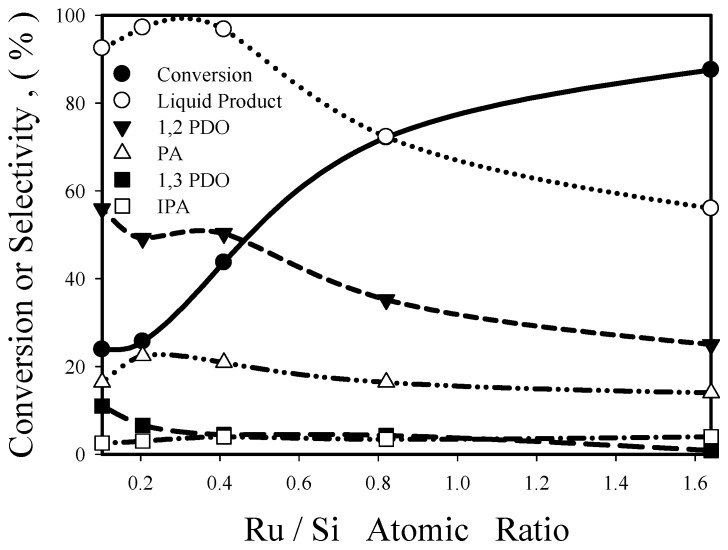
Influence of Ru/Si atomic ratio on glycerol conversion and product selectivity.

Figure 9 presents the relationship between glycerol conversion/product selectivity and Ru/Si atomic ratio under the following conditions: temperature = 130 °C, pressure = 1100 psig, glycerol concentration = 40 wt %, reaction time = 8 h, catalyst weight = 0.63 g. With the increase of Ru/Si atomic ratio from 0.1 to 1.6, glycerol conversion increases continuously (from 23.9% to 87.6%), which should be due to the increase of Ru content in the catalyst. But 1,3-PDO selectivity decreases continuously (from 11% to 0.9%) with increasing Ru/Si ratio, suggesting that it is more difficult to remove the middle OH group in the glycerol molecules when the catalyst is crowded with Ru atoms. 1,2-PDO selectivity is around 50% and overall liquid-phase product (the combination of diols and alcohols) selectivity is greater than 92% for Ru/Si atomic ratio ≤0.4, but decrease rapidly after the further increase of Ru/Si ratio. This can be explained in terms of the competitive adsorption between reactant (glycerol, a triol) and liquid-phase products (diols and alcohols). At low Ru/Si ratio and low glycerol conversion, most Ru sites are covered by glycerol molecules because the adsorption strength typically is in the order: triol > diol > alcohol. At high Ru/Si ratio and high conversion, more Ru sites are available for adsorbing liquid-phase products and gaseous products (H_2_, CO or CO_2_) are produced via C-C bond cleavage of the adsorbed diols/alcohols.

Reaction rates (based on unit catalyst weight), −r_A_’, were calculated from conversion data (shown in Figure 9) according to the following equation:

−r_A_’ = moles of glycerol fed × conversion/(catalyst weight × reaction time) (1)

Re-04RuSi catalyst had a reaction rate of 0.017 mol/h·g catalyst at 130 °C and 1100 psig, which is 8.4 times that (0.00206 mol/h·g catalyst at 160 °C and 1160 psia) calculated from the data of Ma and He [15]. That is, Re-Ru@SiO_2_ catalysts exhibit much greater activity than Re-Ru/SiO_2_ catalyst prepared by conventional impregnation method.

Re addition significantly increased catalyst activity and PDO selectivity. Under the reaction conditions of Figure 9, the reaction rate of the Re-04RuSi catalyst was 30% greater than that of the corresponding Ru@SiO_2_ catalyst (without Re addition). The former had a 1,2-PDO selectivity of 50.3% and a 1,3-PDO selectivity of 4.5%, which are better than those (38.9% and 1%, respectively) of the latter. These are consistent with the results of previous papers, which demonstrated that Re addition to Ru, Pt and Ir catalysts enhanced glycerol C–O hydrogenolysis activities and increased 1,3-PDO selectivity compared to the corresponding monometallic catalysts [14,26]. Chia et al. studied cyclic ether hydrogenolysis on Rh-Re catalysts, the combination of their NH_3_ TPD data and density function theory (DFT) calculation results suggested that hydroxyl groups on rhenium atoms are acidic, due to the strong binding of oxygen atoms by rhenium [27]. It is possible that the glycerol hydrogenolysis proceeds by the hydrogenolysis of the alkoxide species on Re with hydrogen species on the Ru metal surfaces, similar to the mechanism proposed by Amada et al. for the hydrogenolysis of 1,2-propanediol to propanols on Rh-ReO*_x_*/SiO_2_ catalysts [28].

**Figure 10:**
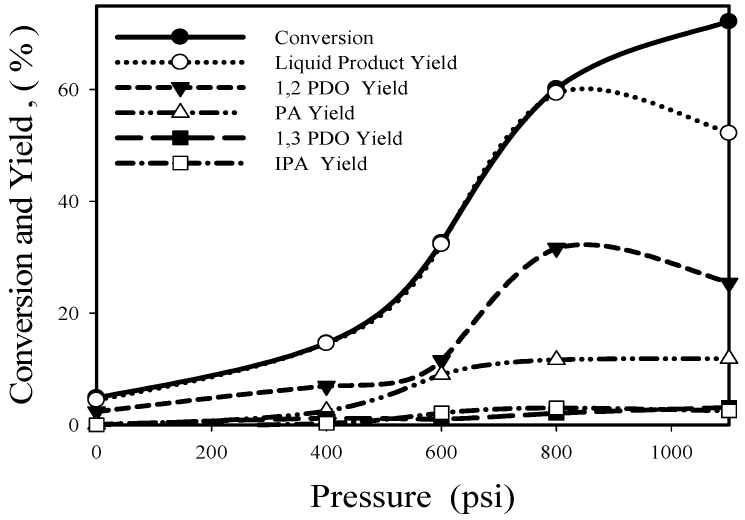
Influence of hydrogen pressure on glycerol conversion and product yield for the Re-08RuSi catalyst.

Figure 10 shows the effect of hydrogen pressure in glycerol hydrogenolysis at 130 °C and 8 h using Re-08RuSi catalyst.

With the increase of pressure from 0 to 1100 psig, glycerol conversion increases from 4.9% to 72.2%, but the maximum 1,2-PDO yield (31.6%) and the maximum yield of overall liquid-phase products (59.3%) occur at 800 psig. The decrease of overall liquid-phase product selectivity (from 98.6% at 800 psig to 72.3% at 1100 psig) should be due to the low glycerol coverage on Ru sites.

The effects of reaction temperature (in a range of 110–160 °C) on glycerol conversion and diol/alcohol selectivies are presented in Table 1 for Re-08RuSi catalyst, which were obtained at 1100 psi, 8 h and 0.63 g Re-08RuSi catalyst. Table 1 shows that glycerol conversion increases with increasing reaction temperature and reaches 100% at 160 °C. However, the selectivities of overall liquid-phase product, 1,2-PDO and 1,3-PDO decrease with increasing reaction temperature and reached 0% at 160 °C. That is, all liquid products were converted to gaseous products (CO, CO_2_ and hydrogen, as identified by GC analyses) at 160 °C and 1100 psi for the Re-08 RuSi catalyst.

It is known that glycerol can be either converted to diols/alcohols via hydrodeoxygenation (based on the pathways shown in Figure 1), or can be used to produce hydrogen through aqueous phase reforming (APR) [29,30,31]. In Table 1, PDO selectivity improved significantly by using low reaction temperature. For example, the decrease of reaction temperature from 130 °C to 110 °C resulted in the increase of 1,2-PDO selectivity from 35.2% to 63.4% and the increase of 1,3-PDO selectivity from 4.3% to 8.5%. The results suggest that the activation energies for PDO conversion to alcohols are higher than those for PDO formation from glycerol. That is, the formers are more temperature sensitive than the latters. At 160 °C, the disappearance of diols/alcohols completely and the generation of gaseous products (CO, CO_2_ and hydrogen) only indicate that APR (C–C bond breaking) favors at higher reaction temperatures [29]. The production of hydrogen through the aqueous-phase reforming (APR) of glycerol is considered as a promising catalytic process and the results of Table 1 indicate that Re-08RuSi catalyst is also a very good catalyst for producing hydrogen at mild reaction condition. In Table 1, methanol selectivity is less than 1% for all conversions, indicating that the O–H bond rupture (hydrogenolysis) is the fundamental prerequisite for C–C cleavage [29]. Re-08RuSi catalyst was reused at 120 °C three times for testing its stability, glycerol conversion decreased slightly from 34.6% (the 1st time use) to 32.8% (the 3rd time use), which indicates that Re-Ru@SiO_2_ catalyst is stable under the glycerol hydrogenolysis conditions.

Kinetic study was carried out with Re-08RuSi and Re-04RuSi catalysts. To determine reaction rate parameters, the following differential equation was established to describe the reaction system in a batch reactor by assuming a pseudo-first-order rate equation for glycerol (denoted as A) hydrogenolysis:

−dC_A_/dt = k C_A_ (2)

Integration of Equation (2) yields

ln(1 − X) = kt(3)

**Figure 11:**
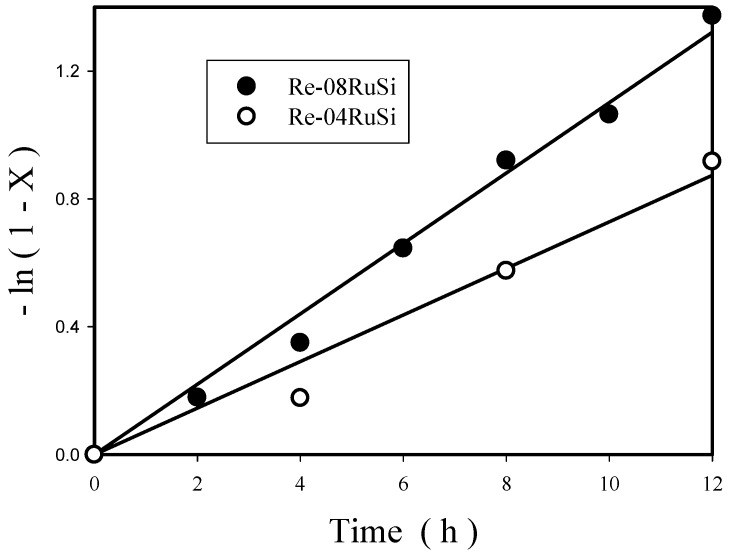
Test of pseudo-first-order kinetic model for Re-04RuSi and Re-08RuSi catalysts at 130 °C and 800 psig.

Experimental results of Re-08RuSi and Re-04RuSi catalysts at 130 °C and 800 psig were plotted according to Equation (3), and a straight line passing through zero was obtained, as illustrated in Figure 11. Therefore, the rate of glycerol disappearance is first-order with respect to glycerol concentration. The rate equation (based on unit volume of reaction solution) can be written as

−r_A_ = k C_A_ (4)

with

k = A exp(−E/RT)(5)

**Figure 12:**
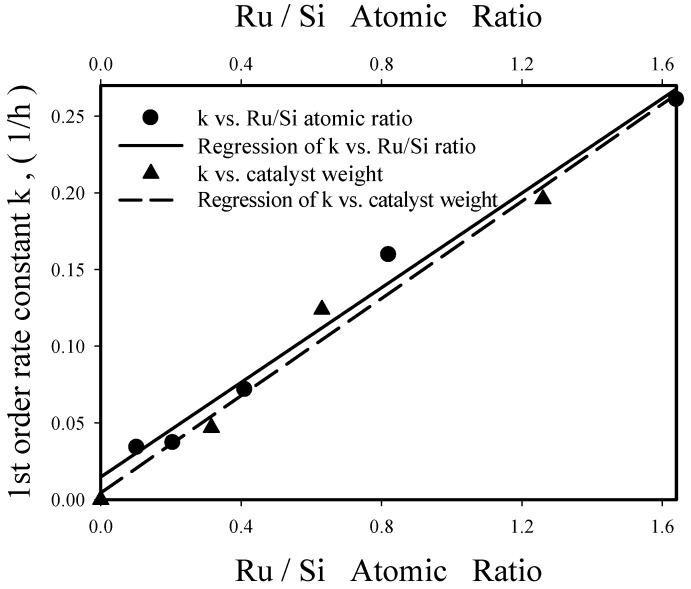
Pseudo-first-order rate constant k (at 130 °C and 800 psi) versus Cu/Si atomic ratio and Re-08RuSi catalyst weight.

The rate constants k at 130 °C obtained from Figure 11 are 0.11/h and 0.072/h for Re-08RuSi and Re-04RuSi, respectively. The higher rate constant obtained with the former (Re-08RuSi) should be mainly due to its greater Ru content, as shown in Figure 12, which presents the effects of Ru/Si atomic ratio and Re-08RuSi catalyst amount on rate constant k. It is interesting to note that k increases linearly with the increase of Ru/Si atomic ratio and with the increase of Re-08RuSi catalyst amount, suggesting that nearly all Ru sites have identical activity for catalyzing glycerol hydrogenolysis.

For the Re-08RuSi catalyst, frequency factor A and activation energy E were 1.06 × 10^13^/h and 107.8 kJ/mol, respectively, which were obtained from an Arrhenius plot of lnk versus 1000/T. In the region of pressure ≥800 psig, rate constant increased linearly with hydrogen pressure (a straight line passing through zero was obtained by plotting k versus pressure), which suggests that glycerol hydrogenolysis is first order with respect to hydrogen pressure.

**Figure 13:**
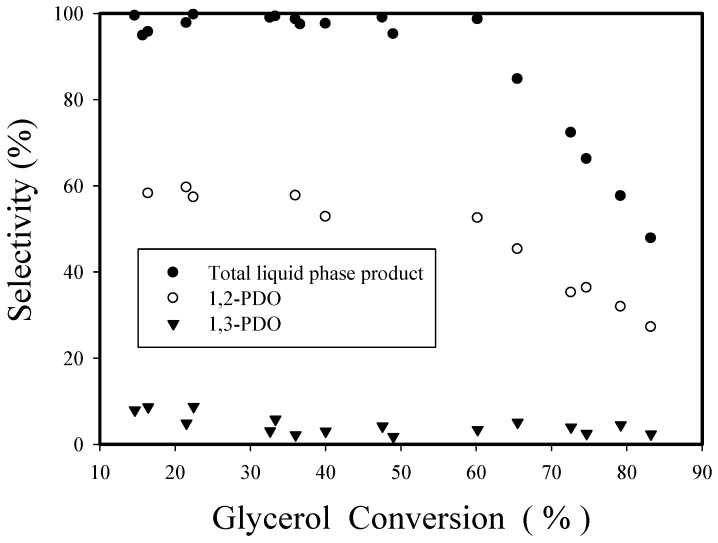
Influence of glycerol conversion on selectivities to overall liquid-phase product, 1,2-PDO and 1,3-PDO for Re-08RuSi catalyst.

Figure 13 shows the effect of glycerol conversion on the selectivities of overall liquid-phase products, 1,2-PDO and 1,3-PDO for Re-08RuSi catalyst, which were obtained at 130 °C using the Re-08RuSi catalyst with the variations of glycerol concentration (20–50 wt %), hydrogen pressure (400–1100 psi), reaction time (2–12 h) and catalyst amount (0.32–1.26 g). All data points essentially fall on a single curve. The selectivities of overall liquid-phase products and 1–2 PDO are not sensitive to the change of glycerol conversion when glycerol conversion is ≤60.2%, but decrease rapidly at the higher conversion range. 1,3-PDO selectivity decreases significantly when glycerol conversion is greater than 30%, suggesting that 1,3-PDO is easier than 1,2-PDO to turn into alcohols. The results are consistent with the activity order (1,3-PDO~glycerol > 1,2-PDO~1-propanol) proposed by Peng et al. for a Pt/Al_2_O_3_ catalyst [30].

Comparisons of Figure 12 (rate constant increases linearly with increasing Ru/Si ratio) and the surface area data (surface area decreases with increasing Ru/Si ratio) indicate that Ru content is much more important than surface area for determining catalyst activity.

## 3. Materials and Methods

### 3.1. Catalyst Preparation

Ru@SiO_2_ nanoparticles with five different Ru/Si atomic ratios were prepared by coating slica onto the surface of Ru-polyvinylpyrrolidone (PVP) colloids, according to the well-known Stober method, which included hydrolysis and condensation of tetraethyl orthosilicate (TEOS) in ethanol, using ammonia as catalyst to initiate the reaction. The Ru-PVP colloids were synthesized by chemical reduction of Ru^3+^ in an alkaline environment using formaldehyde as reducing agent and PVP as protecting agent [17,18]. The calcined Ru@SiO_2_ nanoparticles were then impregnated to incipient wetness with NH_4_ReO_4_ aqueous solution to obtain Re-Ru@SiO_2_ catalysts. The starting materials were RuCl_3_·*x*H_2_O (containing 42 wt % Ru, Seedchem, Melbourne, Australia), NH_4_ReO_4_ (Sigam-Aldrich, St. Louis, MO, USA), polyvinylpyrrolidone (molecular weight = 8000; ACROS, Geel, Belgium), tetraethyl orthosilicate (ACROS), NaOH, NH_4_OH (Showa Chemicals, Tokyo, Japan), formaldehyde (Scharlan Chimie, Barcelona, Spain), ethanol, and acetone (Echo Chemical, Tou Fen, Taiwan).

The main steps for catalyst preparation were as follows [17,18]:

1. Prepare an aqueous solution of RuCl_3_ and PVP, using 2 g RuCl_3_·xH_2_O, 96 mL de-ionized water and 8.2 g PVP.
2. Add 3.3 g formaldehyde and 0.59 g NaOH to the above solution to synthesize Ru-PVP colloids.
3. Wash the Ru-PVP colloids with acetone three times and then dry the products obtained.
4. Prepare and sonicate an aqueous solution containing Ru-PVP colloids, ethanol (213 mL), NH_4_OH (10.9 mL) and de-ionized water (34.4 mL).
5. Add TEOS (amount depends on Ru/Si atomic ratio) and agitate the resulting solution at room temperature for 24 h.
6. Collect the Ru@SiO_2_ nanoparticles by washing, centrifuging, and drying.
7. Calcine the sample at 400 °C for 4 h.
8. Incipient impregnation of 2 g Ru@SiO_2_ particles with an aqueous solution containing 0.2 g NH_4_ReO_4_.
9. Calcine the Re-Ru@SiO_2_ sample at 400 °C for 4 h.
10. Reduce the particles in a gas of 5% hydrogen in 95% argon at a heating rate of 1 °C/min to 200 °C, and maintain at 200 °C for 4 h.

### 3.2. Catalyst Characterization

A Micromeritics surface area analyzer and porosimetry system (model ASAP 2020) was used to determine catalyst-specific surface area and pore size distribution by nitrogen adsorption. A field emission scanning electron microscope (JEOL JSM-7000F) and a transmission electron microscope (JEOL JEM2100F) were used to observe catalyst particle size and morphology. Catalyst crystalline structure was examined by X-ray diffraction (XRD) crystallography on a Shimadzu XRD-6000 diffractometer with Cu Kα radiation. Catalyst reducibility was studied with a temperature-programmed reduction (TPR) method; details are given elsewhere [18].

### 3.3. Reaction Studies

Hydrogenolysis of glycerol to diols/alcohols was carried out with a 600 mL stirred reactor made of stainless steel (supplied by Parr Instruments Co., Moline, IL, USA). In a typical run, 18.5 g (0.2 gmol) glycerol (Alfa Aesar, Ward Hill, MA, USA), 0.63 g Re-Ru@SiO_2_ nanoparticles prepared above, and 27.8 g water (i.e., 40 wt % glycerol aqueous solution) were mixed together and charged into the reactor. The agitator speed was set at 500 rpm, hydrogen was introduced into the reactor at a desired pressure, and the reaction mixture was then heated to the desired temperature. At the end of the reaction, the component compositions were determined with a Shimadzu (Kyoto, Japan) high performance liquid chromatography (model: LC-10A) equipped with a 250 mm long C-18 column and a UV detector (wavelength was set at 254 nm). The glycerol conversion was defined as (moles of glycerol reacted)/(moles of glycerol fed to the reactor) × 100%, product yield was defined as(moles of product obtained)/(moles of glycerol fed to the reactor) × 100%, product selectivity was defined as (moles of product obtained)/( moles of glycerol reacted ) × 100%.

## 4. Conclusions

Re-Ru@SiO_2_ nanoparticles with five different Ru/Si atomic ratios were prepared by coating silica on PVP-stabilized nano-ruthenium colloids, followed by incipient impregnating with NH_4_ReO_4_ aqueous solution. Mesoporous structure was generated due to the burning out of the PVP molecules. The increase of Ru/Si atomic ratio resulted in the increase of catalyst activity but the decrease of pore volume, surface area and particle size. Re addition improved Ru@SiO_2_ performance but retarded RuO_2_ reduction. The rate of glycerol disappearance was first–order with respect to glycerol concentration, and the rate constant (with an activation of 107.8 kJ/mol) increased linearly with increasing Ru/Si atomic ratio, catalyst amount and hydrogen pressure when pressure ≥800 psig. Reaction rate at 130 °C for a Re-Ru@SiO_2_ catalyst (with a Ru/Si atomic ratio of 0.4) was 8.4 times that (at 160 °C) reported previously for a Re-Ru/SiO_2_ catalyst prepared with conventional impregnation method, which was ascribed to the greater Ru content and smaller size of the former. Under isothermal condition, product selectivity correlated well with glycerol conversion. 98.6% overall liquid-phase product selectivity (including 52.5% 1,2-PDO selectivity and 3.5% 1,3-PDO selectivity) at 60.2% glycerol conversion was obtained at 130 °C for the Re-Ru@SiO_2_ catalyst with a Ru/Si atomic ratio of 0.8. The use of lower reaction temperature and higher hydrogen pressure improved PDO selectivity.

## Figures and Tables

**Table 1:** Effects of reaction temperature on glycerol conversion and diol/alcohol selectivities.

Reaction Temp. (°C)	110	120	125	130	160
conversion (%)	27.42	34.59	45.06	72.16	100.00
methanol sel. (%)	0.65	0.36	0.49	0.34	0
isopropanol sel. (%)	2.59	2.19	3.43	3.44	0
ethanol sel. (%)	3.94	5.15	5.83	7.89	0
n-propanol sel. (%)	15.94	19.12	8.18	16.42	0
1.2-PDO sel. (%)	63.36	58.29	64.77	35.16	0
EG sel. (%)	4.50	5.76	6.64	4.72	0
1.3-PDO sel. (%)	8.53	8.05	2.89	4.31	0
Overall liquid product sel. (%)	99.50	98.92	92.23	72.27	0

Reaction conditions: 18.52 g glycerol, 27.78 g water, t = 8 h, P = 1100 psi, 0.63 g Re-08RuSi catalyst.
